# Clinical outcomes of patients with rheumatoid arthritis who underwent percutaneous coronary intervention: A Korean nationwide cohort study

**DOI:** 10.1371/journal.pone.0281067

**Published:** 2023-02-14

**Authors:** Sang Jin Ha, Se-Jun Park, Bora Lee, Hyesung Moon, Bo Young Kim

**Affiliations:** 1 Division of Cardiology, Department of Internal Medicine, Gangneung Asan Hospital, University of Ulsan College of Medicine, Gangneung, Korea; 2 Total Healthcare Center, Kangbuk Samsung Hospital, Sungkyunkwan University School of Medicine, Suwon, Korea; 3 Institute of Health & Environment, Seoul National University, Korea.2 Rexsoft Corp., Seoul, Korea; 4 Rexsoft Corp., Seoul, Korea; 5 Division of Rheumatology, Department of Internal Medicine, Gangneung Asan Hospital, University of Ulsan College of Medicine, Gangneung, Korea; Faculdade de Medicina de São José do Rio Preto, BRAZIL

## Abstract

**Objective:**

Rheumatoid arthritis (RA) increases the risk of cardiovascular disease. This study aimed to investigate the short-and long-term prognosis of patients with and without RA who underwent percutaneous coronary intervention (PCI).

**Methods:**

The Korean National Health Insurance Service claims database was used to extract data on 236,134 patients (34,493 with RA and 201,641 without RA) who underwent PCI between 2008 and 2019. The primary outcome was major adverse cardiovascular events (MACE), including all-cause mortality, myocardial infarction, stroke, transient ischemic attack, or coronary revascularization with short-term (30-day) and long-term outcomes. The secondary outcomes were the individual components of MACE.

**Results:**

During a 10-year follow-up, patients with RA showed a shorter median survival time from MACE than their counterparts (with RA: 4.29 years vs. without RA: 6.10 years). RA was significantly associated with an increased risk of MACEs in long-term outcomes (hazard ratio (HR) 1.07, 95% confidence intervals (CI) 1.06–1.09, *p*<0.001), but not with short-term outcomes (HR 1.02, 95% CI 0.99–1.06, *p* = 0.222). RA was an independent predictor of an increased risk of all the MACE components.

**Conclusion:**

In patients who underwent PCI, RA did not increase the risk of short-term cardiovascular outcomes but increased the risk of long-term adverse outcomes.

## Introduction

Rheumatoid arthritis (RA) is a systemic autoimmune disease characterized by chronic inflammatory polyarthritis. Extra-articular manifestations are also common during the chronic inflammatory state of RA, and cardiovascular disease is one such extra-articular complication. In two meta-analyses, RA was associated with a 48% increased risk of cardiovascular disease and a 50% increased risk of cardiovascular death compared to the general population [1, 2]. The increased risk of cardiovascular disease in patients with RA was comparable to the risk of type 2 diabetes mellitus, a traditional cardiovascular risk factor [3].

Percutaneous coronary intervention (PCI) is the most common revascularization method for patients with coronary artery disease. Studies have reported that patients with RA with acute coronary syndrome have typical angina symptoms less frequently, and patients with RA with myocardial infarction (MI) receive acute reperfusion and secondary preventive medications less frequently than controls [4, 5]. These study results suggest that there may be differences in the clinical characteristics and PCI outcomes of patients with RA undergoing PCI compared to the general population. Despite a higher risk of cardiovascular disease in patients with RA and an increased prevalence of patients with RA undergoing PCI, studies on PCI outcomes in patients with RA are limited and controversial [6–9]. Therefore, this study was aimed at determining whether cardiovascular outcome risk increased in patients with RA in a large population-based PCI cohort over a 12-year period.

## Methods

### Data sources and study population

This retrospective cohort study was conducted using data from the Korean National Health Insurance Service (KNHIS) Database. The database contains patient data including demographics, diagnosis codes of the International Classification of Diseases Tenth Revision (ICD-10), procedure codes, and prescription records. The study was approved by the institutional review board of Gangneung Asan Hospital (Approval number: 2020-04-010-001). The requirement for informed consent was waived because the sample cohort from the KNHIS was provided in an anonymous form for research purposes.

**Fig 1:**
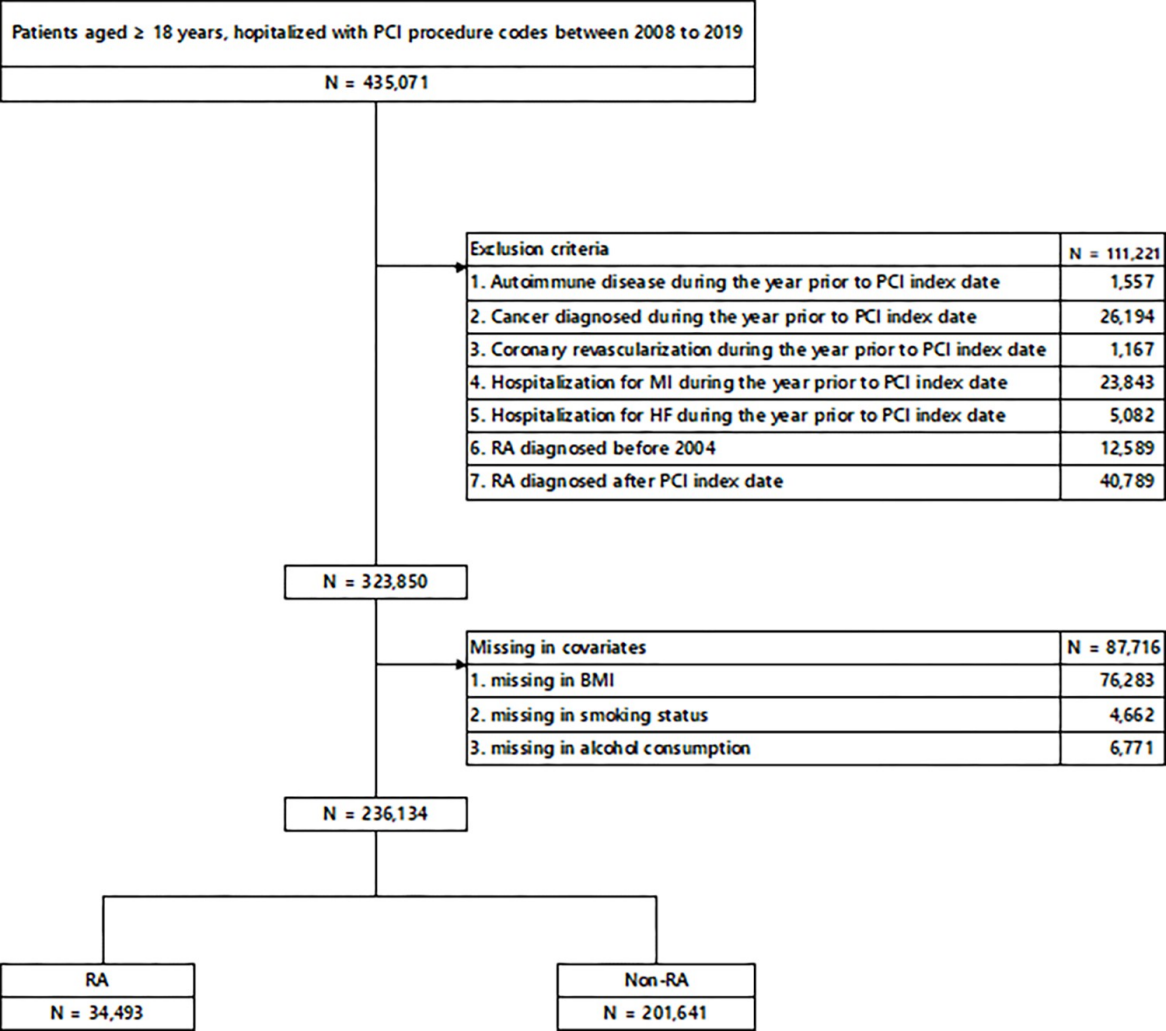
Flow chart of the study population that underwent PCI between 2008 and 2019 in the Korean National Health Insurance Service database. PCI, percutaneous coronary intervention; RA, rheumatoid arthritis; MI, myocardial infarction; HF, heart failure; BMI, body mass index.

We selected patients aged ≥18 years who were hospitalized with PCI procedure codes between January 1, 2008, and December 31, 2019 (Fig 1). A total of 435,071 patients underwent PCI during the study period, and 323,850 patients were identified according to the exclusion criteria. We excluded patients with a diagnosis of autoimmune disease other than RA, including patients with a diagnosis of cancer, those who had undergone coronary artery bypass graft (CABG) or PCI, and those hospitalized for MI or heart failure during the year prior to the PCI index date due to concerns that it may confound cardiovascular outcomes, including all-cause mortality. The index PCI date was defined as the date with no prior coronary revascularization for at least one year before the first procedure date. Patients with RA were selected based on ICD-10 diagnosis codes before the PCI index date. Patients diagnosed with RA before 2004 were excluded when considering the wash-out period for RA diagnosis because the KNHIS database was not properly established before 2003. Patients diagnosed with RA after the PCI index were also excluded. Finally, 34,493 patients with RA were identified in the PCI cohort.

### Comorbidities

Variables were extracted as potential confounders, including basic demographic characteristics, PCI stent type, number of PCI stents, and coronary artery disease at the PCI index date. Cardiovascular risk factors were identified using diagnostic codes (ICD-10), such as hypertension, dyslipidemia, diabetes mellitus, atrial fibrillation, venous thromboembolism, peripheral vascular disease, stroke or transient ischemic attack (TIA), heart failure, chronic obstructive pulmonary disease, and moderate to severe chronic kidney disease. The prevalence of cardiovascular risk factors was defined if the patient had a diagnosis code within six months of the PCI index date. Smoking status was categorized based on current smoking status (nonsmoker, past smoker, or current smoker). Alcohol abuse was defined as heavy drinking in the recommended standards presented by the National Institute on Alcohol Abuse and Alcoholism when the patient’s drinking record was converted to a standard unit [10]. The use of drugs was computed for angiotensin-converting enzyme inhibitors or angiotensin receptor Ⅱ blockers, beta-blockers, anticoagulants, anti-platelet agents, statins, and other lipid-lowering agents within six months of the PCI index date.

### Study end points

We evaluated major adverse cardiovascular events (MACE), which are a composite end-point of all-cause mortality, hospitalized MI, stroke or TIA, or coronary revascularization in short-term (30-day) and long-term outcomes. We also evaluated outcomes, which consisted of individual components of MACE and risk for MACE in prespecified subgroups. All causes of mortality were defined using recorded information from January 1, 2008, to December 31, 2019, in connection with the Korean Statistical Information Service data. MI, stroke or TIA were identified using ICD-10 diagnosis codes. Coronary revascularization was defined as hospitalization for either PCI or CABG identified using inpatient procedure codes.

### Statistical analysis

The t-test and chi-square test were performed to compare continuous and categorical variables, respectively, between patients with and without RA. The Kaplan-Meier survival curves for patients with and without RA were plotted based on the covariates, and the log-rank test was used to confirm statistical significance. For multivariate analysis, Cox proportional hazards regression modeling was performed for factors such as age, sex, body mass index (BMI), current smoking, stroke or TIA, heart failure, hypertension, dyslipidemia, diabetes mellitus, moderate to severe chronic kidney disease, and diagnosis at PCI index date. By using these methods, hazard ratios (HRs) and 95% confidence intervals (CIs) were calculated by using these models. Finally, we conducted subgroup analyses based on age (<65 and ≥65 years), sex, obesity (BMI >30), hypertension, dyslipidemia, diabetes mellitus, current smoking, and indication for PCI (diagnosed with acute coronary syndrome at PCI index date). Age, sex, BMI, current smoking, stroke or TIA, heart failure, hypertension, dyslipidemia, diabetes mellitus, moderate to severe chronic kidney disease, and diagnosis at PCI index date were adjusted in the subgroup analysis.

All statistical tests were set at a two-sided significance level of 0.05. The Statistical Package for SAS (version 9.4; SAS Institute Inc., Seoul, Korea) and R statistical software (version 4.0.3; R Foundation for Statistical Computing, Vienna, Austria) were used to perform the statistical analysis.

## Results

### Baseline characteristics of the study participants

The study initially enrolled 435,071 patients who underwent PCI for ischemic heart disease; 236,134 participants fulfilled the inclusion criteria (Fig 1). The mean age of the study population was 64.5 ± 11.1 years, and the mean follow-up period was 5.64 ± 3.16 years. The baseline characteristics of patients with and without RA are summarized in Table 1.

**Table 1:** Baseline characteristics of the included patients.

Variable	Total	With RA	Without RA	*p*-value
	(N = 236134)	(N = 34493)	(N = 201641)	
Age at index date (years), mean ± SD	64.5 ± 11.1	68.6 ± 10.1	63.8 ± 11.1	<0.001
Sex (female), n (%)	171344 (72.6)	18531 (53.7)	152813 (75.8)	<0.001
BMI (㎏/㎡), mean ± SD	24.7 ± 3.1	24.7 ± 3.1	24.7 ± 3.0	0.251
Current smoking, n (%)	74051 (31.4)	6880 (20.0)	67171 (33.3)	<0.001
Alcohol abuse, n (%)	837 (0.4)	162 (0.5)	675 (0.3)	<0.001
Duration of RA (years), mean ± SD	-	5.7 ± 3.5	-	<0.001
≤ 5 year, n (%)	-	15820 (45.9)		
> 5 year, n (%)	-	18673 (54.1)		
Revascularization before PCI, n (%)				<0.001
No PCI & No CABG	220660 (93.5)	31722 (92.0)	188938 (93.7)	
PCI	1763 (0.8)	306 (0.9)	1457 (0.7)	
CABG	13711 (5.8)	2465 (7.2)	11246 (5.6)	
Both PCI & CABG	-	-	-	
Comorbidities, n (%)				
Hypertension	193649 (82.0)	29744 (86.2)	163905 (81.3)	<0.001
Dyslipidemia	57451 (24.3)	8320 (24.1)	49131 (24.4)	0.3310
Diabetes mellitus	120770 (51.1)	19493 (56.5)	101277 (50.2)	<0.001
Atrial fibrillation	13701 (5.8)	2358 (6.8)	11343 (5.6)	<0.001
Venous thromboembolism	5461 (2.3)	930 (2.7)	4531 (2.3)	<0.001
Peripheral vascular disease	28885 (12.2)	5911 (17.1)	22974 (11.4)	<0.001
Stroke/TIA	26577 (11.3)	4823 (14.0)	21754 (10.8)	<0.001
Heart failure	37222 (15.8)	6418 (18.6)	30804 (15.3)	<0.001
COPD	11917 (5.1)	2303 (6.7)	9614 (4.8)	<0.001
Moderate to severe CKD	2678 (1.1)	520 (1.5)	2158 (1.1)	<0.001
Use of drugs, n (%)				
ACEI/ARB	29539 (12.5)	4508 (13.1)	25031 (12.4)	0.0010
Beta-blocker	176724 (74.8)	25295 (73.3)	151429 (75.1)	<0.001
Anticoagulants	9003 (3.8)	1662 (4.8)	7341 (3.6)	<0.001
Anti-platelets	235857 (99.9)	34456 (99.9)	201401 (99.9)	0.614
Statin	214779 (91.0)	31784 (92.2)	182995 (90.8)	<0.001
Other lipid lowering agents	17749 (7.5)	2676 (7.8)	15073 (7.5)	0.0670

Data are reported as mean ± SD for continuous variables. *p-*value were computed using Student’s t-test or Wilcoxon’s rank-sum test for continuous variables and the chi-squared test or Fisher’s exact test for categorical variables. SD, standard deviation; RA, rheumatoid arthritis; PCI, percutaneous coronary intervention; BMI, body mass index; CABG, coronary artery bypass graft; TIA, transient ischemic attack; COPD, chronic obstructive pulmonary disease; CKD, chronic kidney disease; ACEI, angiotensin-converting enzyme; ARB, angiotensin receptor blocker.

Compared with patients without RA, those with RA (n = 34,493) were older and included more males, but were less likely to be current smokers. Patients with RA had a more frequent history of coronary revascularization and more comorbidities. However, the rates of dyslipidemia was comparable between patients with and without RA.

The procedural characteristics of the PCI index dates are presented in Table 2. With regard to clinical presentation, acute coronary syndrome was two-thirds of the initial presentation, and ST-elevation MI was lower in patients with RA. Most patients underwent PCI using a drug-eluting stent (DES) (94.9%) from a single vessel (87.2%).

**Table 2:** Procedural characteristics of the included patients at PCI index date.

Variable	Total	With RA	Without RA	*p*-value
	(N = 236134)	(N = 34493)	(N = 201641)	
Clinical presentation, n (%)				<0.001
Acute coronary syndrome				
STEMI	67857 (28.7)	8210 (23.8)	59647 (29.6)	<0.001
NSTEMI	20667 (8.8)	2996 (8.7)	17671 (8.8)	0.644
Unstable angina	75844 (32.1)	12156 (35.2)	63688 (31.6)	<0.001
Stable ischemic heart disease	71766 (30.4)	11131 (32.3)	60635 (30.1)	<0.001
Number of implanted stents per person*, n (%)				<0.001
Single vessel PCI	205842 (87.2)	29842 (86.5)	176000 (87.3)	<0.001
Multivessel PCI	22150 (9.4)	3130 (9.1)	19020 (9.4)	0.036
Type of implanted stents*, n (%)				<0.001
Drug-eluting stents	224005 (94.9)	32471 (94.1)	191534 (95.0)	<0.001
Bare metal stents	3310 (1.4)	417 (1.2)	2893 (1.4)	0.001
Both stent types used	677 (0.3)	84 (0.2)	593 (0.3)	0.117

*p-*value were computed using the chi-square test or Fisher’s exact test. RA, rheumatoid arthritis; PCI, percutaneous coronary intervention; STEMI, ST-elevation myocardial infarction; NSTEMI, non-ST-elevation myocardial infarction.

*The number and type of implanted stents were unknown in 8,142 (3.5%) and 8,142 (3.5%) patients, respectively.

### Association of RA and cardiovascular outcomes in patients treated with PCI

The cardiovascular outcomes of the patients with and without RA who underwent PCI are shown in Table 3. The occurence of MACE in the short- and long-term was 24,702 (10.5%) and 118,809 (50.3%), respectively. In the short-term outcomes, there was no difference in MACE between patients with and without RA, despite an increased risk of MI in patients with RA (adjusted HR 1.09, 95% CI 1.03–1.16, *p* = 0.004). However, in terms of long-term outcomes, patients with RA had a higher risk of MACE than patients without RA (crude HR 1.22, 95% CI 1.20–1.24), which was driven by the risk of stroke or TIA (crude HR 1.38, 95% CI 1.35–1.41) and all-cause mortality (crude HR 1.43, 95% CI 1.39–1.47). The association between RA and cardiovascular prognosis remained consistent after adjusting for confounding covariates. RA increased the risks of MI (adjusted HR 1.07, 95% CI 1.04–1.11) and coronary revascularization (adjusted HR 1.05, 95% CI 1.02–1.08) as well as those of stroke or TIA, and all-cause mortality.

**Table 3:** Risk of clinical outcomes in patients with and without RA.

Cardiovascular outcomes	With RA (N = 34493)		Without RA (N = 201641)					*p-*value
	Event (n)	Event rate (%)	Event (n)	Event rate (%)	Crude HR (95% CI)	*p-*value	Adjusted HR (95% CI)	
30-day MACE	4141	12.0	20561	10.2	1.19 (1.15–1.23)	<0.001	1.02 (0.99–1.06)	0.222
Hospitalized MI (%)	1296	3.8	6771	3.4	1.12 (1.06–1.19)	<0.001	1.09 (1.03–1.16)	0.004
Stroke or TIA	1913	5.6	9245	4.6	1.22 (1.16–1.28)	<0.001	0.98 (0.93–1.03)	0.413
Coronary revascularization	461	1.3	2699	1.3	1.01 (0.92–1.12)	0.779	1.05 (0.95–1.16)	0.384
All-cause mortality	950	2.8	4382	2.2	1.27 (1.19–1.36)	<0.001	1.02 (0.95–1.09)	0.64
Long-term MACE	18538	53.7	100271	49.7	1.22 (1.20–1.24)	<0.001	1.07 (1.06–1.09)	<0.001
Hospitalized MI (%)	4370	12.7	26788	13.3	1.03 (1.00–1.06)	0.082	1.07 (1.04–1.11)	<0.001
Stroke or TIA	9379	27.2	45308	22.5	1.38 (1.35–1.41)	<0.001	1.09 (1.07–1.12)	<0.001
Coronary revascularization	6311	18.3	39750	19.7	1.02 (0.99–1.05)	0.146	1.05 (1.02–1.08)	<0.001
All-cause mortality	6799	19.7	32046	15.9	1.43 (1.39–1.47)	<0.001	1.07 (1.04–1.10)	<0.001

Hazard ratios were determined using Cox proportional hazards regression analysis. The adjusted model includes age, sex, body mass index, current smoking, stroke or transient ischemic attack, heart failure, hypertension, dyslipidemia, diabetes mellitus, chronic obstructive pulmonary disease, moderate to severe chronic kidney disease, and diagnosis at percutaneous coronary intervention index date. RA, rheumatoid arthritis; HR, hazard ratio; CI, confidence interval; MACE, major adverse cardiovascular events; MI, myocardial infarction; TIA, transient ischemic attack.

The reference group for each hazard ratio is patients without RA.

**Fig 2:**
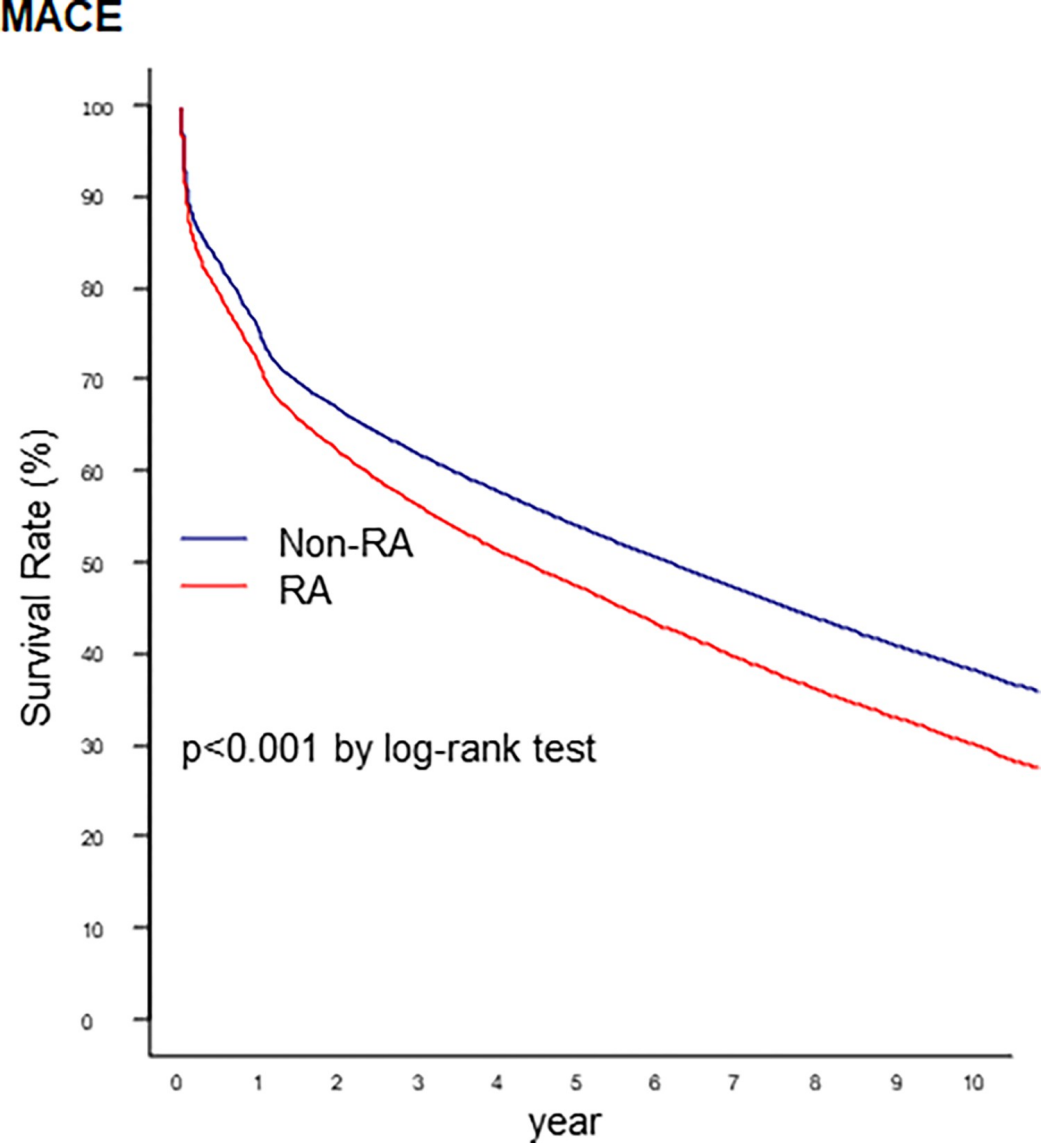
Survival rates from MACE in patients without RA and patients with RA in the PCI cohort. MACE, major adverse cardiovascular events; RA, rheumatoid arthritis; PCI, percutaneous coronary intervention.

**Fig 3:**
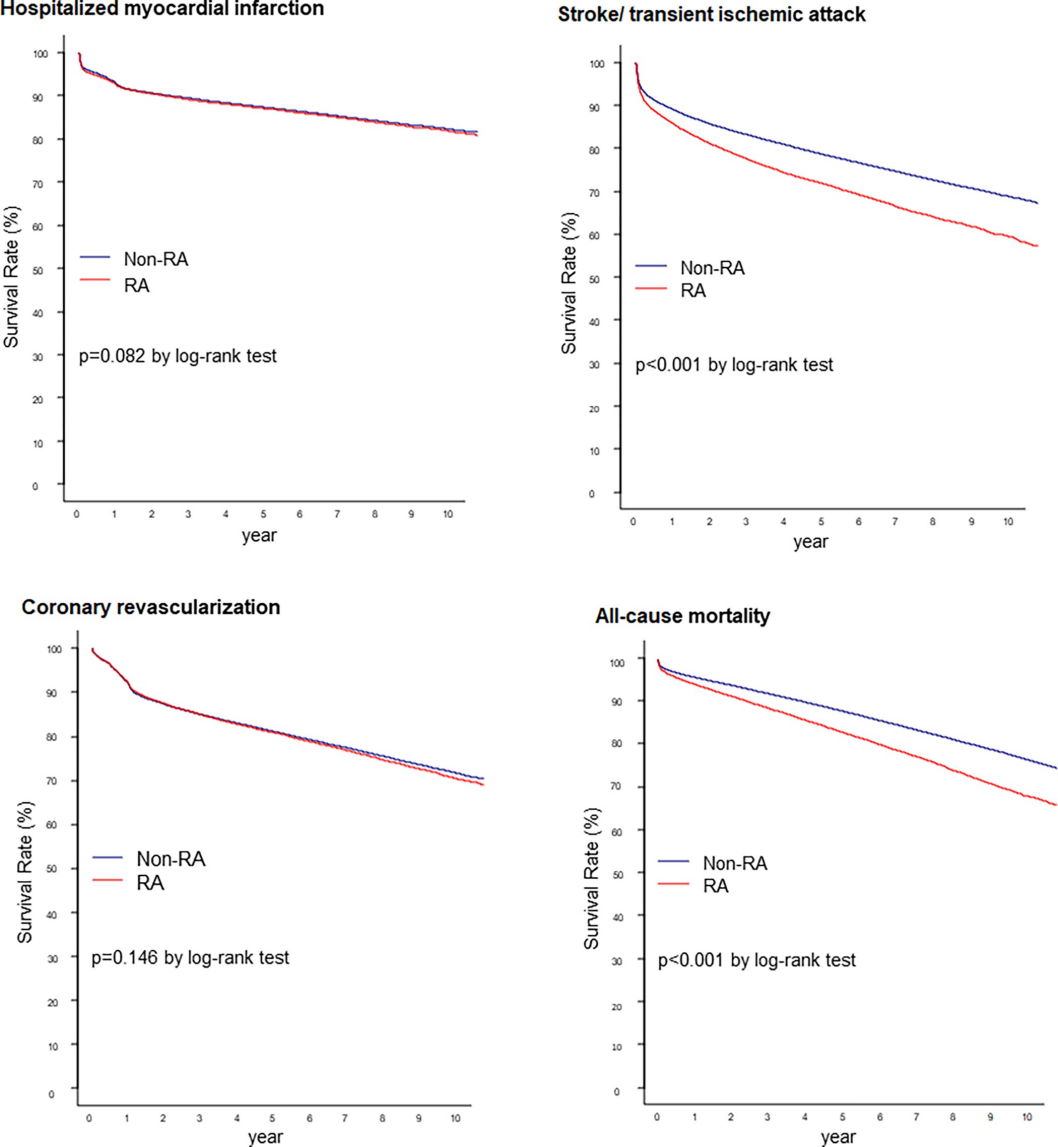
Survival rates from (A) hospitalized myocardial infarction; (B) stroke or transient ischemic attack; (C) coronary revascularization; and (D) all-cause mortality in patients without RA and patients with RA in the PCI cohort. RA, rheumatoid arthritis; PCI, percutaneous coronary intervention.

The median survival time from MACE was 4.29 years (95% CI: 4.16–4.44) in patients with RA and 6.10 years (95% CI: 6.03–6.17) in patients without RA (S1 Table). The Kaplan-Meier survival analysis using MACE is presented in Fig 2. The survival rate was lower in patients with RA than in those without RA (log-rank *p* < 0.001). Among the individual outcomes of MACE, RA decreased the survival rate from stroke or TIA, and all-cause mortality (Fig 3).

### Interaction of RA and MACE in subgroup analysis

**Fig 4:**
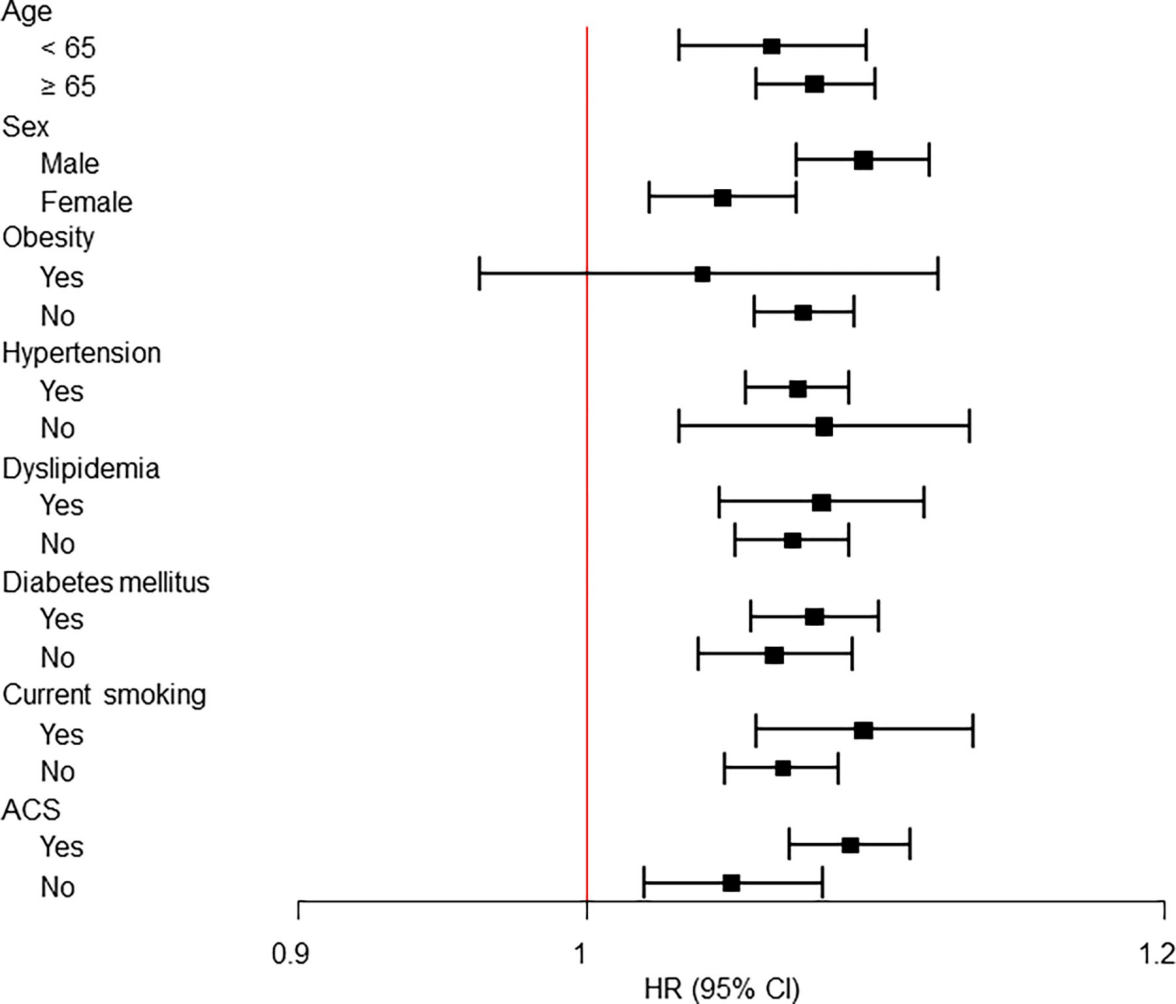
Forest plot for the association between rheumatoid arthritis and the risk of MACE among the subgroups. MACE, major adverse cardiovascular events; ACS, acute coronary syndrome; HR, hazard r.

The association between patients with RA and MACE was assessed by further analysis of selected subgroups based on previous studies of subgroups affecting cardiovascular risk or mortality (Table 4, Fig 4). Non-obese patients with RA had a higher risk of MACE than patients without RA (adjusted HR 1.07, 95% CI 1.06–1.09, *p*<0.001), whereas obese patients with RA had no increase in risk (adjusted HR 1.04, 95% CI 1.00–1.12, *p* = 0.367). Except for obesity, the risk of MACE was higher in patients with RA than in patients without RA, regardless presence of subgroups (Table 4, Fig 4).

**Table 4:** The risk of MACE in patients with and without RA according to the subgroups.

Subgroup		MACE	
		Adjusted HR (95% CI)	*p*-value
Age (years)	< 65	1.06 (1.03–1.10)	<0.001
	≥ 65	1.08 (1.06–1.10)	<0.001
Sex	Male	1.09 (1.07–1.12)	<0.001
	Female	1.05 (1.02–1.07)	<0.001
Obesity	Yes	1.04 (1.00–1.12)	0.367
	No	1.07 (1.06–1.09)	<0.001
Hypertension	Yes	1.07 (1.05–1.09)	<0.001
	No	1.08 (1.03–1.13)	0.001
Dyslipidemia	Yes	1.08 (1.04–1.11)	<0.001
	No	1.07 (1.05–1.09)	<0.001
Diabetes mellitus	Yes	1.08 (1.05–1.10)	<0.001
	No	1.06 (1.04–1.09)	<0.001
Current smoking	Yes	1.09 (1.06–1.13)	<0.001
	No	1.06 (1.05–1.08)	<0.001
Acute coronary syndrome	Yes	1.08 (1.07–1.11)	<0.001
	No	1.05 (1.02–1.08)	0.001

Hazard ratios were determined using Cox proportional hazards regression analysis. The adjusted model includes age, sex, body mass index, current smoking, stroke/transient ischemic attack, heart failure, hypertension, dyslipidemia, diabetes mellitus, chronic obstructive pulmonary, moderate to severe chronic kidney disease, and diagnosis at percutaneous coronary intervention index date. MACE, major adverse cardiovascular events; RA, rheumatoid arthritis; HR, hazard ratio; CI, confidence interval.

The reference group for each hazard ratio is patients without RA.

## Discussion

This study showed that RA affects the long-term outcomes of patients with ischemic heart disease who underwent PCI. The risk of MACE was higher in patients than in those without RA, predominantly caused by stroke or TIA, and all-cause mortality. In terms of short-term prognosis, there were no differences in the MACE risk and survival rates between patients with and without RA. However, there seems to be an obvious decrease in the survival rate from MACE after a year of coronary revascularization. In the subgroup analysis, there was an increased risk of MACE in patients with RA, irrespective of the presence or absence of subgroups, except obesity. Additionally, our study identified differences in clinical characteristics between patients with and without RA who underwent PCI.

The cardiovascular risk in patients with RA was two-fold higher than that in the general population, which was similar to that in patients with type 2 diabetes [3]. Cardiovascular risk increases in RA due to premature atherosclerosis, which is caused by traditional cardiovascular risk factors and factors related to RA, such as chronic inflammation and medications (including glucocorticoids and non-steroidal anti-inflammatory drugs) [11–15]. In particular, chronic inflammation due to RA alters insulin resistance, body composition, and lipid profiles, and these metabolic changes lead to a metabolic syndrome that mediates premature atherosclerosis [16]. In fact, there were studies suggesting that the prognosis for acute coronary syndrome was poor in patients with RA associated with chronic inflammatory status [17, 18]. One study reported that RA predisposes patients to repeat revascularzation at one year, in the absence of methotrexate and tumor necrosis factor-α inhibitors, which is associated with a 50% increased relative risk of repeat revascularization following PCI [19]. These findings highlight the adverse outcomes of chronic inflammation and suggest the importance of anti-inflammatory treatments in patients with RA.

Although most studies have shown consistent results of increased cardiovascular risk in patients with RA, numerous studies on the short- and long-term PCI outcomes in patients with RA have shown controversial results. Previous studies have demonstrated similar short-and long-term outcomes of PCI [6, 20], while a Taiwanese cohort study of 171,547 patients who underwent PCI reported that RA was significantly associated with an increased risk of MACE in the short- and long-term PCI outcomes [7]. In our study, RA was not associated with an increased risk of MACE in short-term outcomes but was associated with a long-term increased risk of MACE, consistent with results from previous studies [8, 21]. The long-term adverse prognosis of PCI in patients with RA is concerning. In our study, it is important to note that the PCI outcome in patients with RA was poor, despite the low rates of acute coronary syndrome, including MI and multivessel PCI stent insertions, compared to patients without RA at the PCI index date. In particular, since the survival rate after a year of PCI is significantly reduced, it is necessary to focus on aggressive secondary prevention. In our subgroup analysis, we found that the risk of MACE was higher in patients with RA than in patients without RA, regardless of the presence of cardiovascular risk factors, except obesity. The increased risk of MACE in patients with RA without obesity may also reflect the rheumatoid cachexia associated with RA. Low or normal body weight may be associated with an increased risk of cardiovascular mortality due to muscle loss caused by inflammatory cytokines in RA [22]. Therefore, our study demonstrates that RA itself may be as important a risk factor that plays a role in determining long-term outcomes after PCI as traditional cardiovascular risk factors, even though RA is not included in the traditional cardiovascular risk scores, such as the Framingham Risk score, and is presented as an atherosclerotic cardiovascular risk enhancer [23, 24].

The strength of our study is the large number of study participants (34,493 in patients with RA) except for other rheumatic diseases, using a large population-based medical record database, despite the low prevalence of RA. Second, we excluded participants with potential confounders that may have confounded cardiovascular outcomes, such as patients hospitalized for coronary revascularization, MI, or heart failure during the one-year pre-index PCI period. Therefore, our study included a large number of patients who underwent first-time PCI, with 93.5% of patients having never undergone previous PCI and CABG. Third, our study also demonstrated that 94.9% of patients underwent PCI with a DES through characterization of the PCI procedure. PCI is indispensable for the treatment of ischemic heart disease. Beyond the era of balloon angioplasty and bare metal stents, the use of DES has become a standard strategy for PCI [25, 26]. Given that the use of DES accounted for 94.9% of cases in this study, the interaction between RA and cardiovascular outcomes might provide clinical insights. DES may reduce the magnitude of the hazard. Compared to the Taiwanese cohort, which included 60% of patients with RA with coronary stents, our study reported a lower risk of all-cause mortality (adjusted HR 1.07 vs 1.55) [7]. Our study had some limitations. First, this study lacked data on the disease activity of RA, RA drugs, and coronary lesions or complexity, which are known to affect cardiovascular outcomes. Second, there may be differences in the prevalence of RA by country and race, and there may be differences in comorbidities related to cardiovascular disease. However, in our study, the participants were limited to Koreans. Third, since this was observational study, we cannot rule out the possibility that there were unmeasured potential confounding variables, despite our efforts to control for confounding variables. Further prospective studies are needed to verify the causal relationship between RA and cardiovascular disease.

In conclusion, RA did not increase the risk of short-term MACE in patients who underwent PCI, but increased the risk of long-term MACE, particularly all-cause mortality and stroke or TIA. Based on the aspects of RA and cardiovascular disease, clinical practice should focus on more detailed and active secondary prevention for a better prognosis.

## Supporting information

S1 Table Survival rates from cardiovascular outcomes in patients with and without RA. (DOCX)
