# Supplementary material for: Clinical outcomes of patients with rheumatoid arthritis who underwent percutaneous coronary intervention: A Korean nationwide cohort study
Source: PLoS One. 2023 Feb 14;18(2):e0281067. doi: 10.1371/journal.pone.0281067 (PMC9928100; doi:10.1371/journal.pone.0281067)
Supplement: S1 Table — (DOCX) [file pone.0281067.s001.docx]

S1 table. Survival rates from cardiovascular outcomes in patients with and without RA

| **Group** | **Median survival time (years) (95% CI)** | **Patients at risk, n / survival rate, %** | |  | |  | |  | |  | |  | |  | | ***p*-value** |
| --- | --- | --- | --- | --- | --- | --- | --- | --- | --- | --- | --- | --- | --- | --- | --- | --- |
|  |  | **Baseline** | **30days** | **1 yr** | | **3 yr** | | **5 yr** | | **7 yr** | | **10 yr** | |  | |  |
| **MACE** | | | | | | | | | | | | | | | | <0.001 |
| Non-RA | 6.10 (6.03-6.17) | 201641/ 99.7 | 181219/ 89.8 | 144124/ 74.7 | 105793/ 61.7 | | 73744/ 53.8 | | 41307/ 47.0 | | 10339/ 38.0 | |  | |  |  |
| RA | 4.29 (4.16-4.44) | 34493/ 99.6 | 30343/ 88.0 | 23038/ 71.1 | 15335/ 56.1 | | 9634/ 47.2 | | 4562/ 39.4 | | 848/ 29.8 | |  | |  |  |
| **Hospitalized myocardial infarction** | | | | | | | | | | | | | | | | 0.082 |
| Non-RA |  | 201641/ 100.0 | 190697/ 96.6 | 172117/ 92.8 | 142448/ 89.4 | | 107458/ 87.3 | | 65598/ 85.2 | | 18836/ 82.2 | |  | |  |  |
| RA |  | 34493/ 100.0 | 32266/ 96.2 | 28388/ 92.5 | 21957/ 89.0 | | 15246/ 87.0 | | 8174/ 85.0 | | 1883/ 81.7 | |  | |  |  |
| **Stroke / transient ischemic attack** | | | | | | | | | | | | | | | | <0.001 |
| Non-RA |  | 201641/ 100.0 | 187763/ 95.3 | 164606/ 89.0 | 131879/ 83.1 | | 95912/ 78.6 | | 56408/ 74.5 | | 15537/ 68.7 | |  | |  |  |
| RA |  | 34493/ 100.0 | 31548/ 94.3 | 26201/ 85.7 | 19024/ 77.4 | | 12446/ 71.8 | | 6264/ 66.3 | | 1314/ 59.4 | |  | |  |  |
| **Coronary revascularization** | | | | | | | | | | | | | | | | 0.146 |
| Non-RA |  | 201641/ 100.0 | 194010/ 98.7 | 168826/ 91.6 | 133777/ 85.0 | | 97707/ 81.1 | | 57333/ 77.4 | | 15453/ 71.7 | |  | |  |  |
| RA |  | 34493/ 100.0 | 32947/ 98.6 | 27983/ 91.9 | 20604/ 84.9 | | 13799/ 80.8 | | 6990/ 76.8 | | 1466/ 70.4 | |  | |  |  |
| **All-cause mortality** | | | | | | | | | | | | | | | | <0.001 |
| Non-RA |  | 201641/ 99.7 | 196509/ 97.8 | 183916/ 95.4 | 156455/ 91.6 | | 119284/ 87.5 | | 73583/ 83.1 | | 21760/ 76.1 | |  | |  |  |
| RA |  | 34493/ 99.6 | 33370/ 97.2 | 30343/ 93.7 | 24057/ 88.1 | | 16803/ 82.5 | | 9055/ 76.9 | | 2124/ 67.7 | |  | |  |  |
| *p-*value was computed using Kaplan-Meier analysis. RA, rheumatoid arthritis; PCI, percutaneous coronary intervention; CI, confidence interval; yr, years; MACE, major adverse cardiovascular events. | | | | | | | | | | | | | | | |  |
